# Unusual manifestation of Erdheim-Chester disease

**DOI:** 10.1186/1471-230X-11-77

**Published:** 2011-06-22

**Authors:** Antony Pan, Terence Doyle, Martin Schlup, Ralf Lubcke, Michael Schultz

**Affiliations:** 1 Gastroenterology Unit, Southern District Health Board, Dunedin, New Zealand; 2 Department of Radiology, Southern District Health Board, Dunedin, New Zealand; 3 Department of Medicine, University of Otago, Dunedin, New Zealand

## Abstract

**Background:**

Erdheim-Chester disease (ECD) is a rare multisystem non-Langerhans cell histiocytosis that is characterized histologically by xanthogranulomatous infiltrates and radiologically by symmetrical sclerosis of long bones. The xanthomatous process is characterized by prominent foamy histiocytes staining positive for CD68, occasionally for PS100 and negative for S100 and CD1a. Gastroenterological involvement is exceedingly rare.

**Case Presentation:**

This case report describes the case of a 69-year-old man who presented otherwise well to the gastroenterology department with unspecific abdominal symptoms, nausea, vomiting and weight loss. ECD involving the gastrointestinal tract was confirmed clinically, radiologically and histologically.

**Conclusion:**

Gastroenterological manifestation of ECD is rare but should be considered in the differential diagnosis in patients presenting with evidence of multi-organ disease and typical radiological features of Erdheim-Chester disease elsewhere.

## Background

Erdheim-Chester Disease (ECD) is a rare multisystem histiocytosis characterized by the xanthomatous or xanthogranulomatous infiltration of tissues with histiocytes, surrounded by fibrosis. The disease can affect multiple organs systems, but gastrointestinal involvement, is exceedingly rare. We describe here the case of a 69-year old man with ECD who presented to the gastroenterology department with unspecific abdominal symptoms, nausea, vomiting and weight loss.

## Case presentation

A previously fit and well 69-year-old man was admitted to the gastroenterology department with a short one month history of lethargy, decreased appetite, persistent vomiting, significant weight loss of six kilograms over 1 month and a dry cough. He denied abdominal pain, haematemesis, dysphagia and a change in bowel habit and described no cardiac, respiratory, neurological symptoms or bone pain. His past medical history includes appendectomy and total hip joint replacement. At the time of admission, he was not on any regular medication.

**Figure 1:**
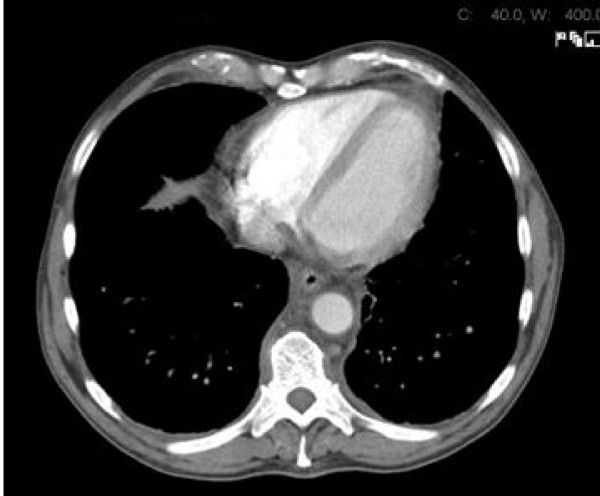
**Axial view of the chest shows presence of peri-aortic and oesophageal infiltration**.

**Figure 2:**
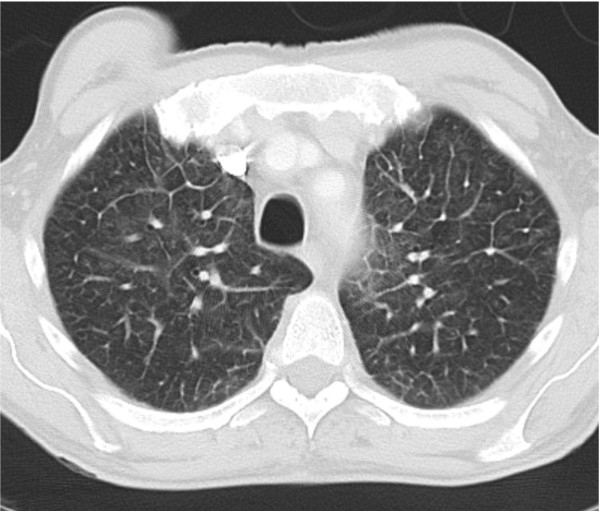
**Imaging of the lung with interstitial thickening of the interlobular septa**.

**Figure 3:**
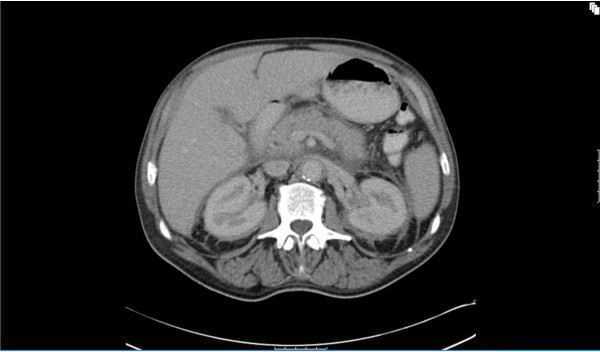
**Axial view shows bilateral and symmetric peri-renal infiltration**. Irregular bands present along posterior parts of peri-renal infiltration gives the appearance of "hairy kidney". The posterior margin of the pancreas is indistinct due to the infiltrate in the retroperitoneum.

Physical examination revealed that he was febrile (38.8C°) and appeared cachexic but with no peripheral stigmata of chronic liver disease. His abdominal examination revealed hepatomegaly but no other organomegaly, per rectal examination was unremarkable. His cardiopulmonary examinations were unremarkable apart from bilateral pitting edema up to his ankles. Peripheral blood analysis revealed an anaemia of chronic disease with hemoglobin of 108 g/L (norm - male 130 - 175 g/L), serum iron 2 μmol/L (norm 10 - 30 μmol/L), transferrin 1.4 g/L (2 - 3.5 g/L), transferrin saturation 6% (norm 16 - 50%) and ferritin 354 μg/L (norm 20 - 500 μg/L). Consistent with a systemic illness the c-reactive protein was increased to 208 mg/L (norm < 5 mg/L) but multiple urine and blood cultures were negative for bacterial infection. Normal serum electrolytes and creatinine of 83 μmol/L (norm 50 - 120 μmol/L). Liver function test was normal apart from a marked hypoalbuminaemia of 28 g/L (norm 35 - 50 g/L). Protein electrophoresis, Vitamin B12, folate, and thyroid functions were normal. Chest X-ray showed interstitial infiltration involving both lung bases. Lung function test was consistent with a restrictive lung disease (FVC 3.33 L (73%), FEV1 2.49 L (72%), FEV1/FVC 75% and decreased in diffusion capacity (DLCO 17.81 ml/min/mmHg). Ultrasound of the abdomen confirmed a 19 cm hepatomegaly, with normal liver texture and no evidence of a mass lesion. Due to ongoing vomiting and weight loss, a gastroscopy was performed with no significant pathology. Computer tomography (CT) of the chest showed peri-aortic tissue infiltration creating the appearance of "coated aorta" (Figure 1) as well as thickening of the interlobular septa in both lungs (Figure 2). The infiltration processes also involved the pulmonary vessels, pericardium, lung parenchyma and the oesophagus. CT of the abdomen showed hypo-attenuated homogenous tissue infiltration with weak contrast enhancement in the renal fossa. Symmetric and bilateral infiltration of the peri-renal, anterior and posterior para-renal spaces gave the appearance of "hairy kidney" (Figure 3). Inferior vena cava, aorta and mesentery were also involved. These findings were seen as consistent with a possible diagnosis of Erdheim-Chester Disease (ECD) [1-3].

**Figure 4:**
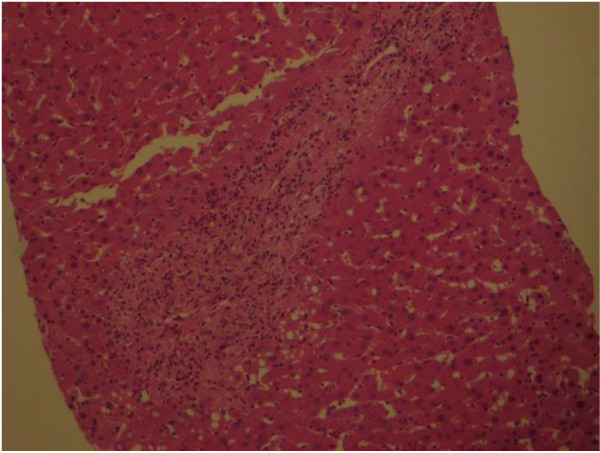
**Histology of the liver showed a predominantly histiocytic (foamy histiocytes) infiltrate**.

**Figure 5:**
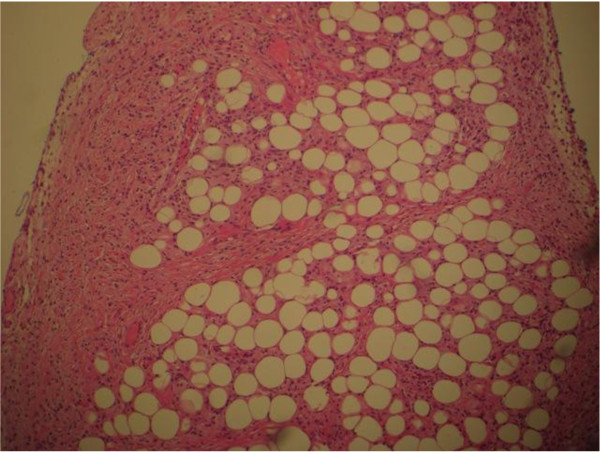
**Biopsy of omentum showed histiocytic infiltrate**.

**Figure 6:**
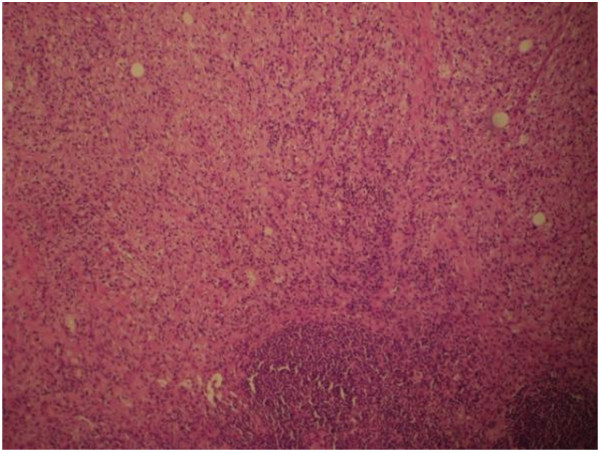
**Biopsy of mesenteric lymph nodes showed evidence of perinodal histiocytic infiltrate**.

A CT guided biopsy of the retroperitoneal space and a bone marrow aspiration were performed which were inconclusive, consistent with reactive inflammatory processes. To confirm the diagnosis of ECD a laparotomy was performed to obtain tissue for diagnostic purposes. Operative findings revealed an infiltrating process involving the liver, omentum and small bowel mesentery. Biopsies were taken from the liver (Figure 4), the omentum (Figure 5) and mesenteric lymph nodes (Figure 6). Histological examination of the tissue showed xanthomatous infiltration involving the liver, omentum and surrounding the lymph nodes. The xanthomatous process was characterized by prominent foamy histiocytes, admixed with occasional giant cells and chronic inflammatory cells. Immunohistochemical stains revealed that these cells were positive for CD68 and negative for S100 and CD1a. There was no evidence of malignancy and the findings allowed a diagnosis of severe multi-organ ECD [1].

**Figure 7:**
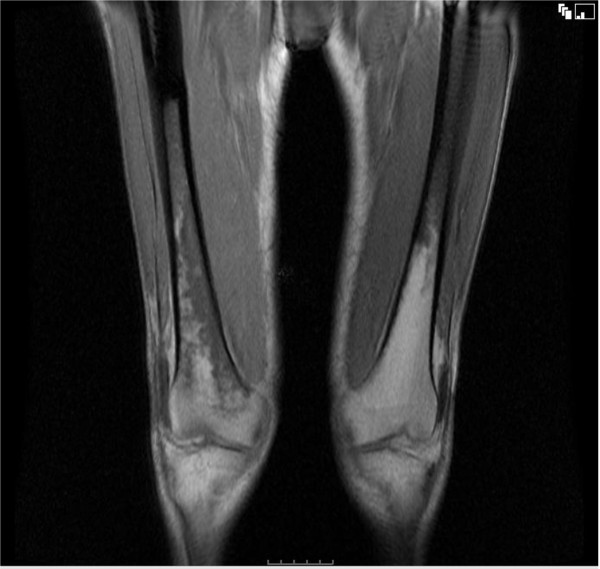
**MRI scan showed an increased marrow signal in the distal femur and proximal tibia**.

Subsequent magnetic resonance imaging (MRI) (Figure 7) of the whole body showed an increased marrow signal in the distal femur and proximal tibia consistent with ECD [4]. Due to further rapid decline, the patient was started on total parenteral nutrition, IV hydrocortisone at 1 g once daily for 2 days, then 500 mg once daily for 8 days before changing to oral prednisone at 100 mg (1 mg/kg/day) for a week. The prednisone dose was then tapered down to 30 mg once daily. Although the patient's appetite improved after the initiation of steroid treatment, hypoalbuminaemia persisted and thrombocytopenia developed. One month after initiation of treatment the patient died of respiratory failure.

## Conclusion

Erdheim-Chester Disease (ECD) is a rare multisystem non-Langerhans cell histiocytosis first described by Jakob Erdheim and William Chester in 1930 [5] and since then approximately 400 cases have been reported [6]. It is characterized by the xanthomatous or xanthogranulomatous infiltration of tissues with foamy histiocytes, surrounded by fibrosis. The aetiology of the disease remains unknown. The disease can affect multiple organs systems, including musculoskeletal, central nervous, cardiac, pulmonary and renal systems. Gastrointestinal involvement, despite being seen more often in other histiocytoses [7], is exceedingly rare and to our knowledge there are 2 case reports regarding involvement of the liver and biliary system [4,8].

Two criteria, of which one should be fulfilled, were proposed by Veyssier-Belot [1] in 1996 as a requirement for the diagnosis of ECD. (1) Typical histological findings with foamy histiocytes nested among polymorphic granuloma and fibrosis or xanthogranulomatosis with CD68-positive and CD1a-negative immunohistochemical staining. (2) Typical skeletal findings with a) radiographs showing bilateral and symmetric osteosclerosis of the diaphyseal and metaphyseal regions in the bones and/or b) symmetric and abnormally increased labeling of the distal ends of the long bones of the lower limbs, and sometimes the upper limbs, on 99Tc bone scintigraphies.

There are typical radiological and pathological features which can lead to the diagnosis, but the clinical spectrum shows a broad variation, ranging from asymptomatic tissue infiltration to fulminant multisystem organ failure.

Bone pain is the most common presenting symptom of ECD mainly affecting the legs, especially knees and ankles and has been reported in 47-86% of patients with ECD [1,9-12]. About half of all patients have extra-skeletal manifestations including retroperitoneal fibrosis, orbital infiltration, interstitial lung disease [2,13], bilateral adrenal involvement [14], testis infiltration [15], breast, central nervous [9,16,17] and/or cardiovascular system [18]. Other general symptoms such as fever, weight loss and weakness can also occur.

Retroperitoneal involvement is secondary to infiltration of the fat and surrounding structures by histiocytes and associated fibrosis. This process can lead to peri-renal and/or ureteral obstruction causing renal impairment. This is reported to occur in 29-59% of patients with ECD [1,19]. Erdheim-Chester infiltration is distinguished from retroperitoneal fibrosis principally by its many foamy histiocytes, lack of plasma cells, and lack of vasculitis [20].

The cardiovascular manifestations of ECD have been known to exist since the disease was first described; this occurs in about 40% of the patients with peri-aortic "fibrosis" as the most frequent cardiovascular involvement. Other manifestations include heart failure, valvular dysfunction, reno-vascular hypertension and pericarditis [18]. It was previously thought that only about 20% of patients have lung involvement however, a recent study reported clinical and/or radiological pulmonary involvement in up to 59% of patients [2]. Usually patients present with dyspnea or a dry cough [2]. Most patients have mediastinal infiltration, diffuse interstitial infiltrates and pleural and/or interlobar septal thickening best seen on high-resolution CT [3]. Characteristic lung histopathology includes the accumulation of histiocytes with variable amounts of fibrosis and a variable lymphoplasmacytic infiltrate in a lymphangitic distribution [13].

The involvement of liver, pancreas, mesentery and gastrointestinal tract is extremely rare [8,4]. The diagnosis of ECD in our patient was challenging as he presented with unspecific symptoms such as a decrease in appetite, persistent vomiting, weight loss and fever. He had features of malnutrition with cachexia, peripheral oedema secondary to hypoalbuminaemia. Evidence of both hepatic and mesenteric involvement of ECD was present, with operative findings of liver, omentum and small bowel mesentery infiltration. Liver biopsy showed xanthomatous infiltration with prominent foamy histiocytes positive for CD68 and negative for S100 and CD1a. Biopsies also confirmed the involvement of mesenteric lymph nodes and the omentum. It was interesting to see that a normal liver function test does not preclude the involvement of the liver by histiocytic infiltration in ECD. Our patient also showed features consistent with skeletal involvement with MRI demonstrating increased marrow signal in the distal femur and proximal tibia. Although bone pain is the most common presenting of ECD, he remained asymptomatic from his skeletal lesions. Other features of ECD demonstrated in our patient include retroperitoneal, cardiovascular and respiratory involvement.

Various therapies for ECD have been proposed, including corticosteroids [1], multiple chemotherapeutic regimes including vinblastine, vincristine, cyclosphosphamide, doxorubicin [1], cladribine [1,21-23], radiotherapy [19,24], cyclosporine and alpha interferon [1]. The latter treatment has gained some attention recently with the initial report of a durable response in three patients [25]. This was followed by a series report of treatment with interferon-alpha in eight patients with variable response. While treatment with interferon-alpha is promising, mechanisms of action are still largely unkown [26]. Prognosis of the disease depends largely on the extent and distribution of the extra-skeletal, in particular cardio-vascular [26] and central nervous system [6] involvement. Based on these results it was suggested by the authors that interferon-alpha is considered as a first-line treatment for patients with ECD but a 40% mortality in the first 40 months after diagnosis has still to be accepted [26].

In summary, we describe the case of a 69-year-old man who presented to the gastroenterological department with unspecific symptoms of fever, fatigue, decreased appetite, persistent vomiting and weight loss. He had clinical, radiological and histological features consistent with gastrointestinal involvement of ECD. He also demonstrated the involvement of skeletal, retroperitoneal, cardiovascular, and respiratory system. He fulfilled the two criteria proposed by Veyssier-Belot^1 ^in 1996 for the diagnosis of Erdheim-Chester disease. Although a bone scan was not performed, the MRI STIR showed an increased marrow signal in the distal femur and proximal tibia. Even though bone pain is the most commonly presenting symptom in ECD, apart from general unspecific symptoms, our patient did not show any evidence of bone pain. Despite the multiple extra-skeletal manifestations seen radiologically, gastroenterological manifestation of ECD is rare but should be considered in the differential diagnosis in patients presenting with evidence of multi-organ disease and typical radiological features of Erdheim-Chester disease elsewhere.

## Competing interests

The authors declare that they have no competing interests.

## Authors' contributions

AP was involved in the treatment of the patient, consulted the literature and drafted the manuscript. MS and RL participated in the design of the study and analysis of the results. MS, corresponding author was supervising the study, conducted the literature review and assisted in the writing of the manuscript. All authors read and approved the manuscript.

## Author information

Dr Pan is an advanced trainee in gastroenterology and general medicine at the Dunedin Hospital, Southern District Health Board, New Zealand.

## Pre-publication history

The pre-publication history for this paper can be accessed here:

http://www.biomedcentral.com/1471-230X/11/77/prepub
